# Comparison of Uptake and Prices of Biosimilars in the US, Germany, and Switzerland

**DOI:** 10.1001/jamanetworkopen.2022.44670

**Published:** 2022-12-02

**Authors:** David L. Carl, Yannic Laube, Miquel Serra-Burriel, Huseyin Naci, Wolf-Dieter Ludwig, Kerstin N. Vokinger

**Affiliations:** 1 Institute of Law, University of Zurich, Zurich, Switzerland; 2 Epidemiology, Statistics, and Prevention Institute, University of Zurich, Zurich, Switzerland; 3 Department of Health Policy, London School of Economics and Political Science, London, England; 4 Drug Commission of the German Medical Association, Berlin, Germany

## Abstract

**Importance:**

Biologics account for a substantial proportion of health care expenditures. Their costs have been projected to reach US $452 billion in global spending by 2022. Given recent expiration of patent protection of biologics, a shift toward greater follow-on competition among biosimilars would be expected that would allow greater uptake and lower drug costs.

**Objective:**

To assess uptake and prices of biosimilars in the US compared with 2 European countries (Germany and Switzerland) with national mechanisms for drug price negotiation.

**Design, Setting, and Participants:**

In this cohort study, biologics and biosimilars that were approved in the US, Germany, and Switzerland until August 2020 were identified. Prices and sales data were extracted from public and commercial databases for the years 2011 to 2020. Data were analyzed from August 1, 2021, to February 28, 2022.

**Main Outcomes and Measures:**

Descriptive statistics were used to show temporal trends in the uptake of biosimilars and relative prices compared with those of reference products (ie, biologic agents) for each country. Descriptive analysis was also performed to compare the uptake of biosimilars between the 3 countries limited to biologics that have biosimilars on the market in all countries. To test if biosimilar awareness in each country increased over the last decade, a linear least squares regression was applied.

**Results:**

The study cohort included 15 biosimilars and 6 biologics for the US, 52 biosimilars and 15 biologics for Germany, and 28 biosimilars and 13 biologics for Switzerland. Uptake of biosimilars increased over time in all countries. On average, the biosimilar market share at launch was highest in Germany; however, it increased at the fastest rate in the US. Monthly treatment costs of biosimilars in the US were a median of 1.94 (IQR, 1.78-2.44) and 2.74 (IQR, 1.91-3.46) higher than corresponding costs in Germany and Switzerland, respectively.

**Conclusions and Relevance:**

The findings of this cohort study suggest that more biosimilars have been marketed in Germany and Switzerland than in the US. Policies that counter anticompetitive practices in the US could allow biosimilars to enter the market sooner and could also lower health care costs with improved access. Awareness of biosimilars should be promoted to increase uptake of biosimilars globally.

## Key Points

**Question:**

How do uptake and prices of biosimilars in the US compare with 2 European countries (Germany and Switzerland) with national mechanisms for drug price negotiation?

**Findings:**

This cohort study found that fewer biosimilars entered the market in the US between 2011 and 2020 compared with Germany and Switzerland and on average, the biosimilar market share at launch was highest in Germany yet increased at the fastest rate in the US. Monthly treatment costs of biosimilars were substantially higher in the US compared with Germany and Switzerland.

**Meaning:**

These findings suggest that policies aimed against anticompetitive practices could allow biosimilars to enter the US market more quickly and could result in overall lower costs and that awareness of biosimilars should be promoted to increase uptake of biosimilars globally.

## Teaser

This cohort study compares uptake and prices of biosimilars in the US with those of Germany and Switzerland and assesses whether biosimilar awareness increased in each country over the last decade.

## Introduction

Biologic drugs are complex molecules derived from living cells.^1^ Since the approval of the first biologic, recombinant human insulin, in 1982, the number of biologics has greatly increased, accounting for over one-third of new medicine approvals.^2,3^ Biologics for cancer and other disorders account for a substantial proportion of health care expenditures.^4^ In the US, they make up only 2% of prescriptions but account for 37% of net drug spending.^1,5^ Biologics account for the fastest growing segment of pharmaceutical research and development and have been projected to reach US $452 billion in global spending by 2022.^6,7^

As the patent protection and regulatory exclusivity periods on biologic drugs expire, biologics face competition from a follow-on class of agents known as biosimilars.^4^ A biosimilar is a biologic agent that is not chemically identical but highly similar to an approved biologic agent (also referred to as a *reference product*), with no meaningful differences in efficacy, safety, or purity.^4,8^ An increasing number of biosimilars may spur competition and lower prices, as with generic drugs,^9,10^ and several countries have developed policies to incentivize market entry of biosimilars. For example, in the US, the Biological Price Competition and Innovation Act was implemented in 2010 to create an abbreviated pathway for biosimilars.^11,12^ The pathway was tailored to address distinct properties of the biologic products, which in contrast to small-molecule drugs are large, complex molecules reduced through living systems rather than chemical processes.^13^ The goal was to streamline the regulatory approval process for biosimilars and potentially lower health care costs through competition.^12^ In the European Union, a similar approval pathway was introduced in 2005.^14^

The past several years have been characterized by the expiration of patent protection and regulatory exclusivity periods of biologic drugs. Therefore, a shift toward greater biosimilar competition would be expected that would allow greater uptake and lower drug costs. However, studies have shown that there are varying policies and biosimilar uptake in European countries^15,16^ and that the observed levels of competition and uptake have not reached the expected levels in the US.^1,11,17^ Policy makers in the US and Europe are considering legislative and regulatory reforms intended to promote the competition of biosimilars. To assist such ongoing discussions, we aimed to assess and discuss the uptake and prices of biosimilars in the US compared with Germany and Switzerland, 2 European countries with national mechanisms for drug price negotiation.

## Methods

This cohort study used public, nonidentifiable data that did not constitute research with human participants and was therefore exempt from institutional review board approval and the need for informed consent in accordance with US Department of Health and Human Services section 45 CFR 46.102. The study followed Strengthening the Reporting of Observational Studies in Epidemiology (STROBE) reporting guideline.

### Data Sources and Extraction

Using publicly available databases, we identified all biologics (ie, reference products) and biosimilars that were approved in the US, Germany, and Switzerland through August 2020.^18,19,20,21^ We collected the following information: active ingredient, brand name, and approval date.

For the US, we qualified drugs as biosimilars if they were approved via the 351(k) abbreviated licensure pathway.^12^ For Germany and Switzerland, we considered drugs biosimilars if the European Medicines Agency (EMA) qualified these drugs as such in their public assessment reports. The qualification of drugs as biosimilars is not always congruent in the US and Europe. For example, drugs with the active ingredient enoxaparin or terparatide are considered biosimilars by the EMA (eTable in Supplement 1) but have not been approved via the 351(k) pathway in the US. In such cases, we applied the policies of the respective jurisdiction—ie, we considered drugs with the active ingredients enoxaparin or terparatide as biologics and biosimilars in Germany and Switzerland, but not in the US. Furthermore, we excluded active ingredients if one of the follow-up drugs with the same active ingredient was not approved via the 351(k) pathway in the US or as a biosimilar by the EMA. For example, filgrastim was not approved via the 351(k) pathway, thus we excluded the reference product and biosimilars with the active ingredient filgrastim. Another example is somatropin, which is not considered a biosimilar by the EMA; thus, we excluded the reference product and biosimilars with the active ingredient somatropin. Additionally, we only included drugs if at least one biosimilar had been marketed (ie, if the IQVIA database provided sales data). For example, adalimumab was launched in 2002 in the US,^22^ and although 5 adalimumab biosimilars have been approved to date, none have been marketed owing to patent dispute settlement.^22^

Prices were extracted for the period January 1, 2011, through December 31, 2020. Wholesale acquisition costs were obtained from online drug pricing databases for the US,^23^ Germany,^24^ and Switzerland.^25^ In case of confidential rebates for biologics in Switzerland, list prices were obtained. We extracted quarterly sales volume data for the same period from the IQVIA database for the drugs in our cohort for the US, Germany, and Switzerland.^26^

### Statistical Analysis

Using previously described methods,^27,28^ we calculated monthly treatment costs for each drug using dosing information in the US Food and Drug Administration (FDA)-approved label. For drugs available in multiple strengths, we calculated the lowest monthly treatment costs. All costs were reported in US dollars, applying the exchange rate of October 2020. Descriptive statistics were used to show temporal trends in the uptake of biosimilars and relative prices compared with the reference products for each country.

For each quarter, we calculated the relative market share (uptake) and the relative prices of all biosimilars compared with the reference product (biologic) that shared the same active ingredient. Uptake was defined as the total units of the active ingredient sold by biosimilars divided by the sum of the total units of the active ingredient sold by biosimilars and the reference product at some time point.

To assess the difference in the uptake and relative prices over time between the US and the 2 European countries, we used locally estimated scatterplot smoothing on a subset of our data consisting of observations at launch and the first 4 quarters after launch of active substances for which the drugs were approved and qualified as biosimilars in all 3 countries.

In a separate analysis, we assessed whether there was an association between the uptake of biosimilars and the year of launch for each country to test if biosimilar awareness increased over the last decade. We regressed the market share 1 year after launch on the launch year adjusted again for changes in relative market size and relative prices using ordinary least squares.

Data were analyzed from August 1, 2021, to February 28, 2022. All statistical analyses were performed in R, version 4.1.0 (R Project for Statistical Computing) using ggplot 2 (version 3.3.5) for plots and statistics (version 4.1.0) and using sandwich (version 3.0-1) and stargazer (version 5.2.2) for regression analysis. Two-sided *P* < .05 was considered statistically significant.

## Results

Our study cohort included 15 biosimilars and 6 reference products for the US, 52 biosimilars and 15 reference products for Germany, and 28 biosimilars and 13 reference products for Switzerland. Five substances (enoxaparin sodium, filgrastim, teriparatide, insulin glargine, and insulin lispro) were excluded from the US sample because they were not approved via the FDA’s approval pathway for biosimilars, and 2 biosimilars (adalimumab and etanercept) were approved but not included because they have not entered the market due to legal disputes.

### Uptake of Biosimilars

**Figure 1.**
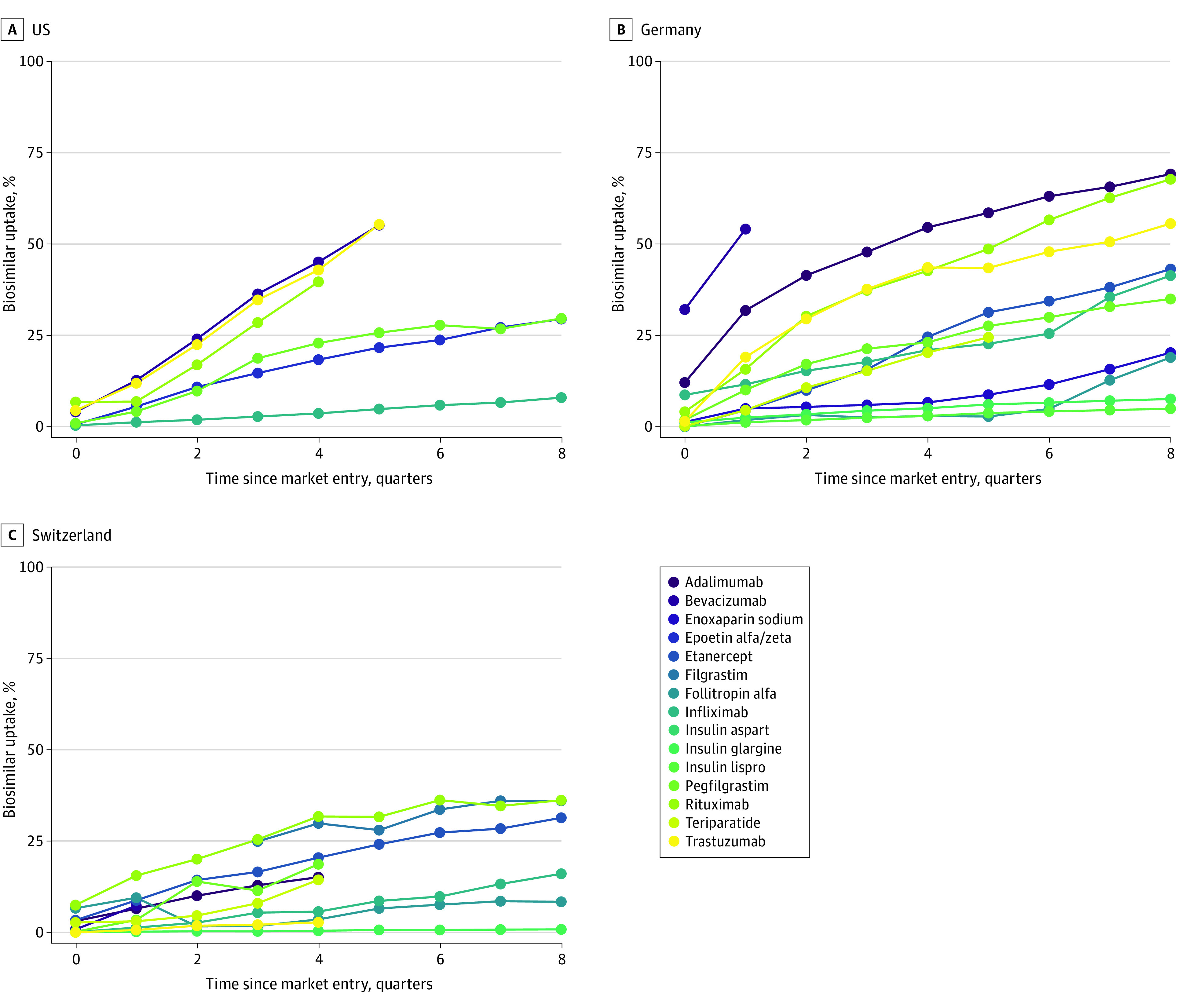
Uptake of Biosimilars in the US, Germany, and Switzerland Each graph represents all biosimilars for each active ingredient.

Uptake of biosimilars increased in all 3 countries over time. However, differences in uptake were observed between active ingredients within and across countries (Figure 1). In the US, uptake was highest for bevacizumab (36%) and lowest for infliximab (3%) 1 year after market entry. Uptake in Germany was highest for adalimumab (48%) and lowest for insulin lispro (2%), whereas in Switzerland, uptake was highest for rituximab (25%) and lowest for insulin glargine (1%) 1 year after market entry (Figure 1).

When comparing the uptake of the 6 ingredients with at least 1 biosimilar marketed in all 3 countries, we found that on average, biosimilar market share was highest in Germany followed by the US and Switzerland. However, biosimilar market share in the US grew at a faster rate compared with Germany and Switzerland.

Biosimilars in the US that entered the market more recently were estimated to experience a faster uptake (as measured by the market share 1 year after launch), whereas in Germany and Switzerland the coefficients were small and not significant (Table).

**Table.** Results of Regression Analyses to Estimate the Association Between Biosimilar Uptake and Year of Launch in the US, Germany, and Switzerland

	Market share 1 y after launch					
	United States		Germany		Switzerland	
	Estimate (95% CI)	*P* value	Estimate (95% CI)	*P* value	Estimate (95% CI)	*P* value
Year of launch	0.15 (0.05 to 0.25)	.01	0.02 (−0.07 to 0.11)	.30	−0.01 (−0.05 to 0.03)	.29
Change in relative market size since launch	0.21 (−1.20 to 1.62)	.29	−0.64 (−2.11 to 0.83)	.21	0.09 (−1.24 to 1.42)	.44
Relative price of biosimilar to reference product	0.44 (−0.37 to 1.25)	.07	−0.70 (−1.90 to 0.49)	.15	−0.17 (−1.61 to 1.26)	.40
Constant	0.63 (0.38 to 0.88)	.004	0.40 (0.08 to 0.72)	.05	0.10 (−0.11 to 0.30)	.18
Observations	6	NA	11	NA	10	NA

Abbreviation: NA, not applicable.

### Prices and Monthly Treatment Costs

**Figure 2.**
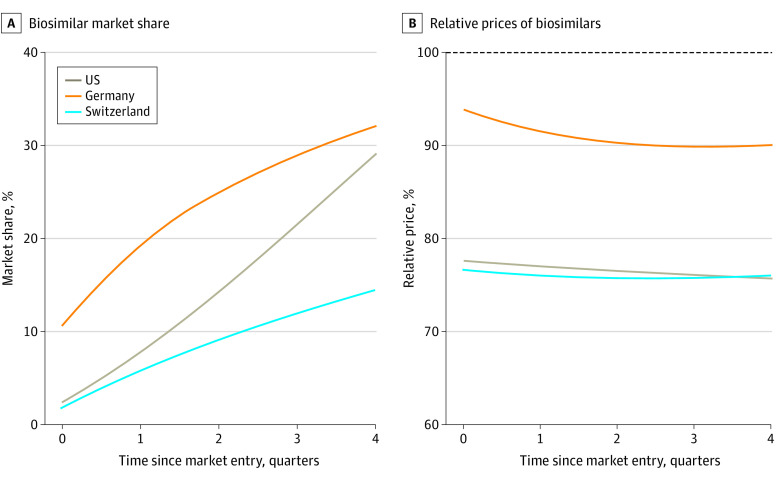
Relative Prices of Biosimilars vs Reference Products (Biologics) in the US, Germany, and Switzerland Each graph represents the average of all biosimilars for each active ingredient.

In the US, the relative prices of biosimilars were lower at market entry compared with their reference products, with a variation between 55% and 90% and a slight decrease over time. In Germany, biosimilars with the active ingredient trastuzumab and pegfilgrastim entered the market with prices higher than their reference products, and the other biosimilar groups entered the market with relative prices between 65% and 100%. In Switzerland, prices for biosimilars were lower at market entry compared with their reference products, with relative prices ranging between 70% and 80%. The relative prices of biosimilars with the active substances pegfilgrastim and enoxaparin sodium decreased over time, whereas the relative prices of other biosimilar groups were stable compared with their reference products (Figure 2).

**Figure 3.**
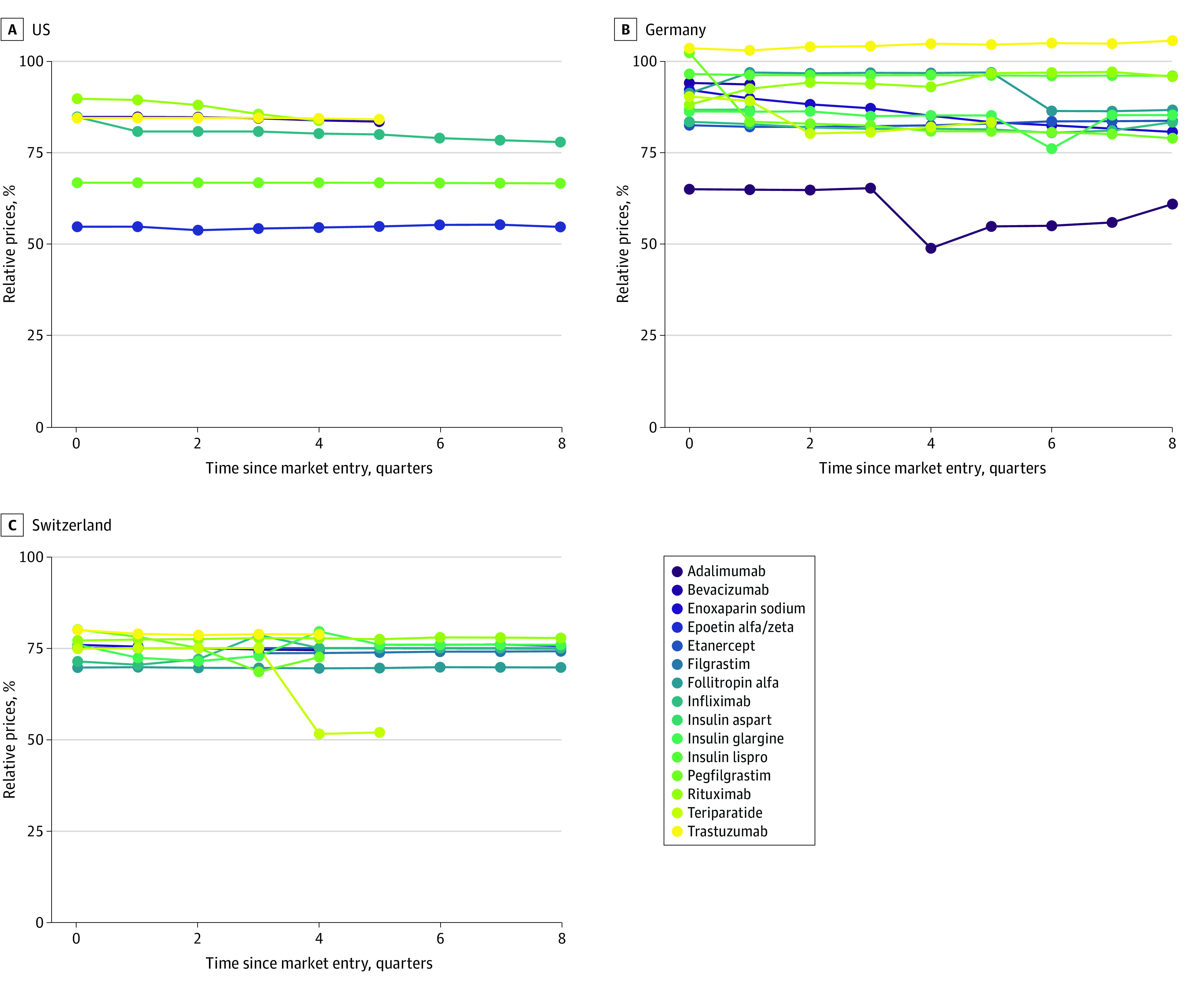
Relative Market Share and Relative Prices of Biosimilars vs Reference Products (Biologics)

In the subset of 6 active ingredients that included at least 1 biosimilar marketed in all 3 countries, relative prices were similar in the US and Switzerland and highest in Germany (Figure 3).

**Figure 4.**
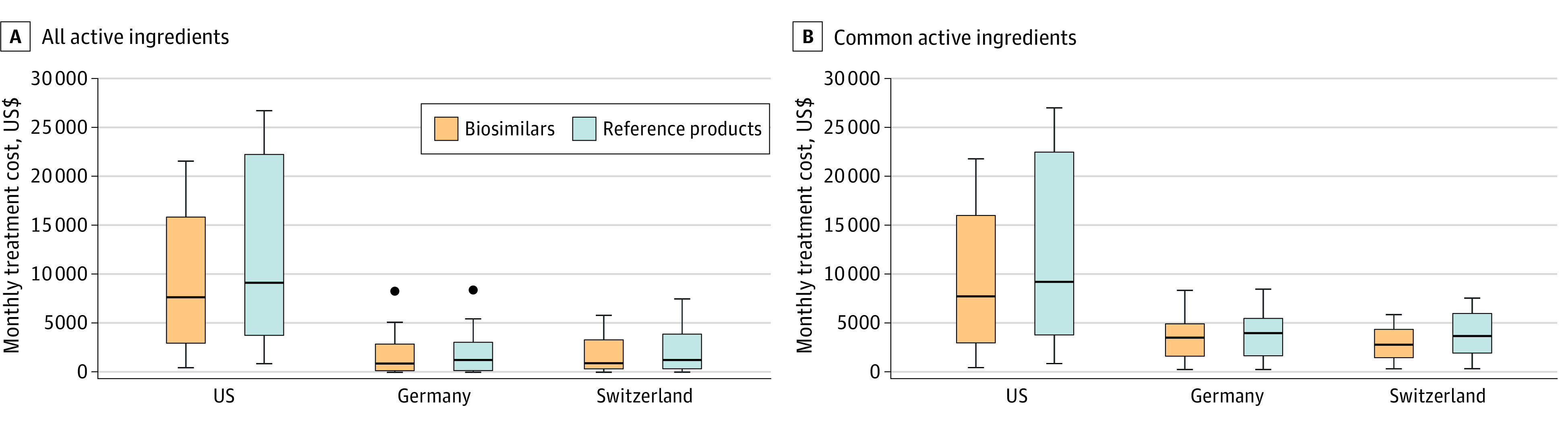
Monthly Treatment Costs of Biosimilars and Reference Products (Biologics) in the US, Germany, and Switzerland A, Monthly treatment costs for all active ingredients. B, Monthly treatment costs of common active ingredients in all 3 countries. Horizontal lines represent the median; boxes represent the IQR; whiskers represent the highest and lowest observation at 1.5 × IQR; dots represent outliers.

Monthly treatment costs of biosimilars in the US were a median of 1.94 (IQR, 1.78-2.44) and 2.74 (IQR, 1.91-3.46) higher than the corresponding costs in Germany and Switzerland, respectively. In the US, median monthly treatment costs per patient as of October 2020 were US $8987 and US $11 503 for the included biosimilars and reference products, respectively. In Germany, median monthly treatment costs per patient were US $932 for biosimilars and US $1285 for reference products, and US $1351 for biosimilars and US $1801 for reference products in Switzerland (Figure 4).

## Discussion

A larger number of biosimilars have been marketed in Germany and Switzerland compared with the US despite comparable approval rates. Uptake of marketed biosimilars increased over time in all 3 countries, and the prices of biosimilars and reference products were substantially higher in the US compared with Germany and Switzerland.

One possible reason for the limited availability of biosimilars in the US could be ongoing patent litigation or agreements to defer entry as a result of settling patent disputes.^11,29,30^ Reference products with the active ingredients bevacizumab, infliximab, or rituximab were protected by a median of 90 patents.^30^ Another example is adalimumab. The originator drug, Humira, was launched in 2002 in the US.^22^ Humira is the top revenue-generating drug in the US, generating US $15 billion in sales in the US alone in 2019.^31^ To date, 5 adalimumab biosimilars have been approved, yet none have been marketed owing to patent dispute settlement.^22^ Such extensive patenting of biologics incentivizes confidential litigation settlements in which manufacturers of biosimilars agree to not market their products years after FDA approval and after market entry in the European Union.^30^ Such exclusionary contracts may result in higher health care costs and delayed availability of biosimilars to patients.^30^ Additionally, the World Health Organization (WHO) Fair Pricing Initiative highlights the importance of timely entry into the market as a key element to improve uptake of biosimilars.^32^

Previous studies have highlighted that limited biosimilar availability in the US might result in skepticism among physicians and patients relating to the efficacy and safety of biosimilars.^29^ For example, a national survey of US physicians found that more than half did not believe that biosimilars were safe and appropriate for use in patients.^33^ However, consistent with a previous study,^15^ the findings of the present study showed an association between launch year and biosimilar uptake; biosimilars introduced more recently in the US market were estimated to have a stronger uptake compared with biosimilars introduced earlier, an observation that was not made for Germany or Switzerland. These results could imply, among other things, an increasing awareness of biosimilars in the US. However, especially in the US, the association was estimated based on a limited number of drugs, and it cannot be predicted whether future biosimilars will experience a higher uptake.

In all 3 countries, relative prices of biosimilars were lower at market entry compared with their reference products, with 2 exceptions in Germany (biosimilars with the active ingredient trastuzumab and pegfilgrastim). The prices of biosimilars compared with their reference products varied more widely in the US (between 55% and 90%) and Germany (between 65% and 103%) compared with Switzerland (between 70% and 80%). The results for Switzerland can be explained with the direct price link policy between biosimilars and their reference product—that is, that the maximum price of a biosimilar is based on a percentage of the originator price (in Switzerland, 25% lower than the reference product).^32,34^ By contrast, Germany does not take into consideration the prices of reference products when negotiating the prices of biosimilars.^35^ Such price link policies could be helpful in the other countries to lower health care costs. However, the WHO highlighted that such fixed discounts could also discourage price competition between different manufacturers that might otherwise lead to greater price reductions.^32^ Other policies have been proposed to enable more competition and lower prices such as tendering or demand-side measures by increasing knowledge about biosimilars among patients, prescribers, and dispensers.^35^

Monthly treatment costs of biosimilars in the US were a median of 1.94 (IQR, 1.78-2.44) and 2.74 (IQR, 1.91-3.46) higher than corresponding costs in Germany and Switzerland, respectively. These results are consistent with former studies that demonstrated higher drug prices in the US compared with European countries.^27,28^ This difference might be explained by the fact that European countries have more comprehensive mechanisms for drug price assessment and negotiation than the fragmented system in the US.^28^

### Limitations

Our study has some limitations. First, our sample size was limited, which resulted in uncertainties in the statistical analysis. Second, we could only include sales data that were provided by IQVIA. This is a limitation because some data was not reported for unknown duration and thus sales data might be underreported. Third, we focused on list prices in the US and did not account for rebates.^36^ Additionally, comparative conclusions between the US and the European Union are limited because the same drugs entered the market at different points in time, and both jurisdictions have either not approved all drugs to date or have not qualified the same drugs as biosimilars.

## Conclusions

This study found that slightly more biosimilars have been marketed in Germany and Switzerland compared with the US. On average, biosimilar market share at launch was highest in Germany but increased at the fastest rate in the US. Prices of biosimilars and reference products were substantially higher in the US compared with Germany and Switzerland. Policies for drug pricing negotiations in the US against anticompetitive practices of exclusionary contracts could allow biosimilars to enter the market sooner and at lower costs, which could result in lower health care costs and improved patient access. Awareness of biosimilars should be promoted to increase the uptake of biosimilars in all three countries.

## Notes

Supplement 1. **eTable.** Monthly Treatment Costs and Year of First Market Entry for Biosimilars in the US, Germany, and Switzerland

Supplement 2.
Data Sharing Statement
