# Supplementary material for: Comparison of Uptake and Prices of Biosimilars in the US, Germany, and Switzerland
Source: JAMA Netw Open. 2022 Dec 2;5(12):e2244670. doi: 10.1001/jamanetworkopen.2022.44670 (PMC9719051; doi:10.1001/jamanetworkopen.2022.44670)
Supplement: Supplement 1. — eTable. Monthly Treatment Costs and Year of First Market Entry for Biosimilars in the US, Germany, and Switzerland [file jamanetwopen-e2244670-s001.pdf]

Supplemental Online Content

Carl DL, Laube Y, Serra-Burriel M, Naci H, Ludwig WD, Vokinger KN. Comparison of uptake and prices of biosimilars in the US, Germany, and Switzerland. *JAMA Netw Open*. 2022;5(12):e2244670. doi:10.1001/jamanetworkopen.2022.44670

eTable. Monthly Treatment Costs and Year of First Market Entry for Biosimilars in the US, Germany, and Switzerland

This supplemental material has been provided by the authors to give readers additional information about their work.

© 2022 Carl DL et al. *JAMA Network Open.*

eTable. Monthly Treatment Costs and Year of First Market Entry for Biosimilars in the US, Germany, and Switzerland

		**Monthly treatment costs**			**Biosimilars first market entry**		
**Active ingredient**	**Biologic (grey), biosimilar (white)**	United States	Germany	Switzerland	United States	Germany	Switzerland
	Humira	-	USD 1,516	USD 1,282	-	-	-
Adalimumab[U]	Amgevita, Imraldi, Hyrimoz, Hulio, Idacio	-	USD 923	USD 954	-	2018	2019
	Avastin	USD 11,954	USD 5,477	USD 4,889	-	-	-
Bevacizumab	Mvasi, Zirabev, Aybintio[U,S]	USD 10,009	USD 5,125	USD 3,667	2019	2020	2020
	Clexane	-	USD 95	USD 152	-	-	-
Enoxaparin[U]	Inhixa, Crusia[S], Hepaxane[S], Enoxaparin Ledraxen[S]	-	USD 86	USD 114	-	2017	2020
	Erypo / Procrit[G,S]	USD 906	USD 309	USD 389	-	-	-
Epoetin alfa / zeta	Epoetin Alfa Hexal[U,S], Abseamed[U], Binocrit[U], Retacrit[S], Silapo[U,S]	USD 497	USD 306	USD 377	2018	2007	2009
	Enbrel	-	USD 1,285	USD 1,228	-	-	-
Etanercept[U]	Benepali, Erelzi, Nepexto[S]	-	USD 933	USD 918	-	2016	2018
	Neupogen	-	USD 3,622	USD 3,925	-	-	-
Filgrastim[U]	Ratiograstim[S], Zarzio, Filigrastim Hexal, Nivestim[S], Grastofil[S], Accofil	-	USD 3,037	USD 3,331	-	2008	2010
	GONAL-f	-	USD 970	USD 989	-	-	-
Follitropin alfa[U]	Ovaleap, Bemfola[S]	-	USD 883	USD 689	-	2014	2018
	Remicade	USD 2,946	USD 1,408	USD 1,801	-	-	-
Infliximab	Inflectra, Remsima[U], Renflexis[S], Zessly[U,S], Avsola[G,S]	USD 2,206	USD 1,273	USD 1,351	2016	2015	2016
	NovoRapid	-	USD 31	-	-	-	-
Insulin aspart[U,S]	Insulin aspart Sanofi	-	USD 27	-	-	2020	-
	Lantus	-	USD 39	USD 42	-	-	-
Insulin glargine[U]	Abasaglar	-	USD 35	USD 32	-	2015	2015
	Humalog	-	USD 31	-	-	-	-
Insulin lispro[U,S]	Liprolog, Insulin lispro Sanofi	-	USD 30	-	-	2017	-
	Neulasta	USD 26,705	USD 5,408	USD 6,322	-	-	-
Pegfilgrastim	Pelgraz[U], Pelmeg[U], Fulphila[S], Ziextenzo, Grasustek[U,S], Cegfila[U,S], Udenyca[G,S]	USD 17,799	USD 4,264	USD 4,584	2018	2018	2019
	Rituxan	USD 25,669	USD 8,420	USD 7,511	-	-	-
Rituximab	Truxima, Rixathon[U], Ruxience[S]	USD 21,564	USD 8,298	USD 5,841	2019	2017	2018
	Forsteo	-	USD 428	USD 379	-	-	-
Teriparatide[U]	Terrosa, Movymia, Teriparatid-Mepha[G]	-	USD 356	USD 197	-	2019	2019
	Herceptin	USD 6,353	USD 2,777	USD 2,472	-	-	-
Trastuzumab	Ontruzant[S], Herzuma[S], Kanjinti, Trazimera, Ogivri[S]	USD 5,359	USD 2,552	USD 1,946	2019	2018	2019

Active substances are not included in the cohort for the following countries or biosimilars are not approved or are not marketed in the following countries:

[U] United States

[G] Germany

[S] Switzerland

© 2022 Carl DL et al. *JAMA Network Open.*
