# Supplementary material for: Comparison of Uptake and Prices of Biosimilars in the US, Germany, and Switzerland
Source: JAMA Netw Open. 2022 Dec 2;5(12):e2244670. doi: 10.1001/jamanetworkopen.2022.44670 (PMC9719051; doi:10.1001/jamanetworkopen.2022.44670)
Supplement: Supplement 2. — Data Sharing Statement [file jamanetwopen-e2244670-s002.pdf]

Data Sharing Statement

Carl. Comparison of Uptake and Prices of Biosimilars in the US, Germany, and Switzerland. *JAMA Netw Open*. Published December 02, 2022. doi:10.1001/jamanetworkopen.2022.44670

Data
Data available: No

Additional Information
Explanation for why data not available: We do not have any patient data.
